# Use of stewardship smartphone applications by physicians and prescribing of antimicrobials in hospitals: A systematic review

**DOI:** 10.1371/journal.pone.0239751

**Published:** 2020-09-29

**Authors:** R. I. Helou, D. E. Foudraine, G. Catho, A. Peyravi Latif, N. J. Verkaik, A. Verbon

**Affiliations:** 1 Department of Medical Microbiology and Infectious Diseases, Erasmus Medical Center, Rotterdam, The Netherlands; 2 Division of Infectious Diseases, Geneva University Hospitals and Faculty of Medicine, Geneva, Switzerland; 3 Department of Medical Sciences, Uppsala University, Uppsala, Sweden; Rabin Medical Center, Beilinson Hospital, ISRAEL

## Abstract

**Background:**

Antimicrobial stewardship (AMS) programs promote appropriate use of antimicrobials and reduce antimicrobial resistance. Technological developments have resulted in smartphone applications (apps) facilitating AMS. Yet, their impact is unclear.

**Objectives:**

Systematically review AMS apps and their impact on prescribing by physicians treating in-hospital patients.

**Data sources:**

EMBASE, MEDLINE (Ovid), Cochrane Central, Web of Science and Google Scholar.

**Study eligibility criteria:**

Studies focusing on smartphone or tablet apps and antimicrobial therapy published from January 2008 until February 28th 2019 were included.

**Participants:**

Physicians treating in-hospital patients.

**Interventions:**

AMS apps

**Methods:**

**S**ystematic review.

**Results:**

Thirteen studies met the eligibility criteria. None was a randomized controlled trial. Methodological study quality was considered low to moderate in all but three qualitative studies. The primary outcomes were process indicators, adherence to guidelines and user experience. Guidelines were more frequently accessed by app (53.0% - 89.6%) than by desktop in three studies. Adherence to guidelines increased (6.5% - 74.0%) significantly for several indications after app implementation in four studies. Most users considered app use easy (77.4%—>90.0%) and useful (71.0%—>90%) in three studies and preferred it over guideline access by web viewer or booklet in two studies. However, some physicians regarded app use adjacent to colleagues or patients unprofessional in three qualitative studies. Susceptibility to several antimicrobials changed significantly post-intervention (from 5% decrease to 10% - 14% increase) in one study.

**Conclusions:**

Use of AMS apps seems to promote access to and knowledge of antimicrobial prescribing policy, and increase adherence to guidelines in hospitals. However, this has been assessed in a limited number of studies and for specific indications. Good quality studies are necessary to properly assess the impact of AMS apps on antimicrobial prescribing. To improve adherence to antimicrobial guidelines, use of AMS apps could be considered.

## Introduction

Appropriate prescribing of antimicrobials is crucial for individual patients to increase the chance of therapeutic success and to prevent spread of antimicrobial resistance (AMR) on a broader scale. For this reason, governments and healthcare institutions have developed and implemented antimicrobial stewardship (AMS) programs to improve appropriate prescribing [1–3].

Local antimicrobial guidelines help physicians to prescribe appropriate antimicrobial therapy. However, guidelines change and increasing complexity of care requires easily accessible and frequently updated guidelines. Printed booklets and digital documents may not be sufficient for this purpose. In the age of information technology (IT), many processes within the healthcare system have been digitized or automated and IT has become an intrinsic part of modern medicine [4–6]. IT interventions such as electronic health records (EHR), clinical decision support systems (CDSS), and antimicrobial drug approval systems increase guideline adherent prescribing. Such tools assist in a more timely intravenous to oral switch, and decrease overall antimicrobial consumption [4, 7, 8]. Nevertheless, appropriate prescribing of antimicrobials can still be improved [9, 10]. AMS is important for general practice and hospitals, but prevalence of antimicrobial resistant microorganisms is the highest in hospitals, even in countries with overall low resistance rates [11, 12]. Furthermore, reserve antimicrobials are mainly used in hospitals [11, 12].

With the introduction of smartphones, applications (apps) can be accessed without the necessity of a non-mobile desktop and can simultaneously provide a framework to integrate CDSSs. Besides accessibility, apps offer several other advantages such as the most up to date content, short start-up time and administrator privileges to inform users of specific updates [13].

In Europe and North America the number of unique mobile phone subscribers was respectively 85% and 84% at the end of 2017. In the same year over 318.000 mobile health (mHealth) apps were available in app stores [14, 15]. The majority of healthcare workers utilizes mHealth apps (77.2%) on a regular basis in the United States [16]. Although smartphone apps have high potential for becoming a key component of AMS programs, user experience, uptake and effect on prescription of antimicrobials have not been systematically reviewed to the best of our knowledge. The aim of this study was to systematically review antimicrobial stewardship apps and their impact on prescribing by physicians treating in-hospital patients

## Methods

This systematic review was performed in accordance with the guidelines of Preferred Reporting Items for Systematic Reviews and Meta-Analyses: The PRISMA Statement [17]. (S1 Text).

### Eligibility criteria

Studies which focused on AMS app use by physicians treating in-hospital patients were assessed for eligibility. Studies focusing on smartphone or tablet apps and antimicrobial therapy published from January 2008 until February 28^th^ 2019 were included. The year 2008 was chosen since the two most popular app stores, App Store (iOS) and the precursor of Google Play (Android) were launched that year [18, 19]. We included randomized controlled trials (RCT), non-RCTs, time series, before-after studies and qualitative studies. Excluded were studies solely considering antimicrobial prophylaxis, only including patients younger than 18 years of age, case reports, conference papers, editorials, letters to editor and reviews or meta-analysis. Language was no exclusion criterion. We excluded studies which only described app use in the general practice and outpatient setting. In these settings, prevalence and severity of infectious diseases, available antimicrobials and routes of administration differ significantly compared to an in-hospital setting.

### Search strategy and review design

EMBASE, MEDLINE (Ovid), Cochrane Central, Web of Science and Google Scholar databases were searched for relevant quantitative and qualitative studies published before February 28^th^ 2019. The search strategy was developed together with an information specialist and specified for each database. Search terms included “antimicrobial”, “anti-infective agent”, “prescription”, “application” and “mobile phone” (S2 Text). Search results were imported to Endnote (Clarivate Analytics, Philadelphia, PA, USA). After removal of duplicate studies, two investigators (RH and DF) independently screened all articles on title and abstract. Articles were included for full text review if selected by either investigator. In case of doubt, articles were included for full text review. Both investigators independently assessed full texts for eligibility and extracted data. Disagreements were resolved in discussion with a third investigator (AV).

### Study outcome

Primary study outcomes are process indicators such as number of downloads, average monthly use and guidelines assessed, adherence to guidelines and user experience. Secondary study outcomes are drug consumption, susceptibility and costs.

### Data analysis

Data was extracted using standardized forms (S3 Text). The quality of included studies was independently assessed by two investigators (RH and DF). Disagreements were resolved by discussion with a third investigator (AV). To assess the five different study designs we used five risk of bias assessment tools [20–24]. Due to large variations in study design and outcome parameters, study outcome could not be pooled and used for meta-analysis.

## Results

### Study characteristics

**Fig 1:**
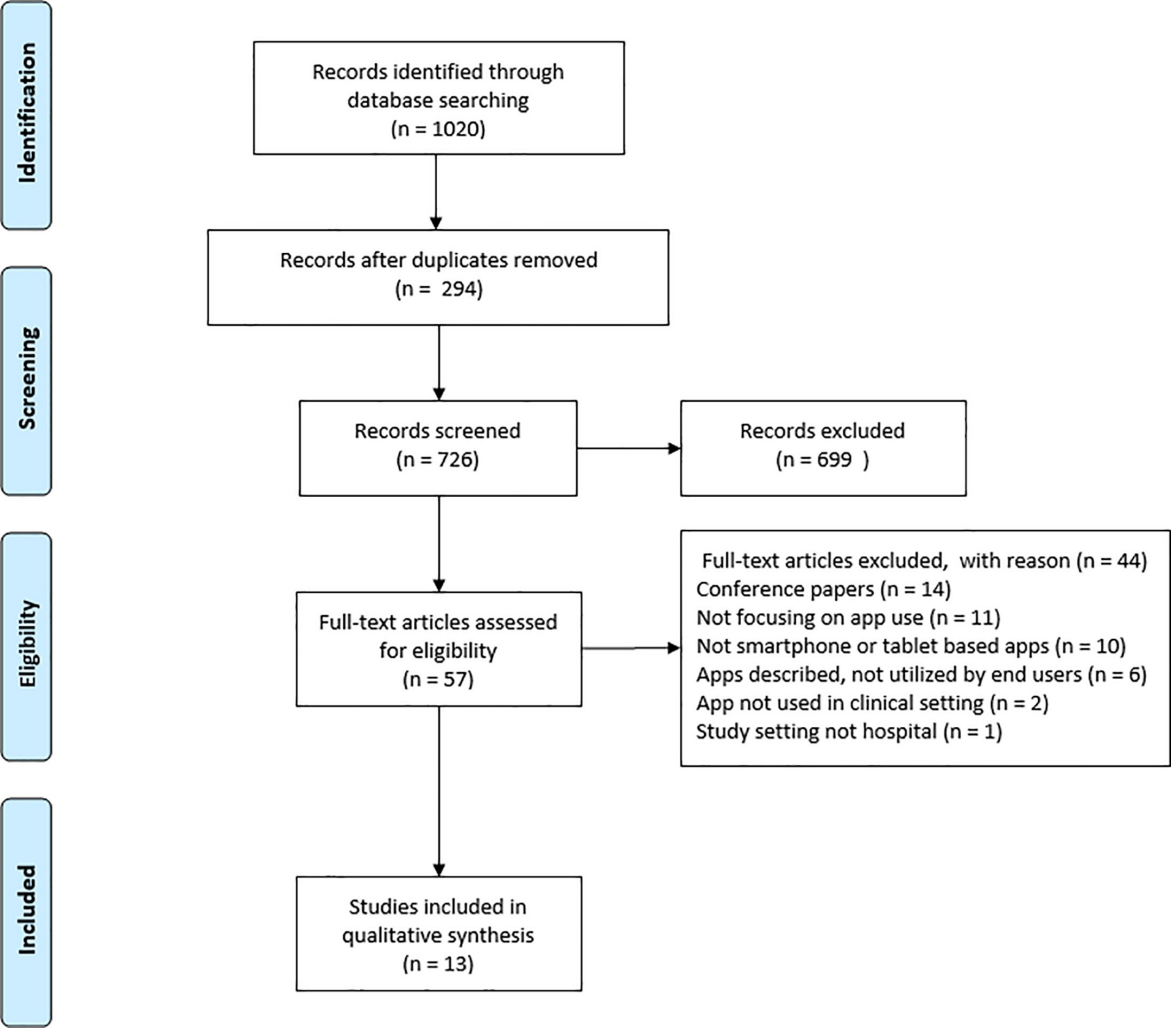
Study selection.

Thirteen studies met the eligibility criteria and were evaluated in this systematic review (Fig 1). Primary outcomes were process indicators such as downloads, average app use and time spent per guideline, evaluated in seven studies. Changes in adherence to guidelines and user experience were analysed in four and five studies, respectively. Antimicrobial consumption was evaluated in two studies (Table 1). In ten of the thirteen studies the app was custom built for the study (Table 2).

**Table 1:** Study characteristics.

Author	Country	Study period	Study design	Setting	Primary outcome	Patients included	
						Pre-intervention	Intervention
Charani (2013)	UK	2011–2012	Cross-sectional, before-after & qualitative	Hospital	Process indicators, user experience	n/a	n/a
Payne (2014)	UK	*N/A*	Before-after & qualitative	Hospital	Process indicators, user experience	n/a	n/a
Panesar (2016)	Canada	2013	Cross-sectional & before-after	Hospital	Process indicators, user experience	n/a	n/a
Blumenthal (2017)	USA	2014–2016	Cross-sectional	Ward	Antimicrobial consumption	148	199
Charani (2017)	UK	2008–2014	Interrupted time series	Hospital	Adherence to guidelines	*N/A*	*N/A*
Fralick (2017)	Canada	2015	Before-after	Ward	Knowledge of prescribing, user experience	n/a	n/a
Haque (2017)	Bangladesh	2015	Before-after	Ward	Adherence to guidelines	325	516
Hoff (2018)	USA	2016–2017	Cross-sectional	Hospital	Process indicators	n/a	n/a
Tuon (2017)	Brazil	2014–2015	Before-after	Hospital	Antimicrobial consumption, susceptibility and cost, process indicators	n/a	n/a
Shenouda (2018)	UK	*N/A*	Qualitative	Hospital	User experience	n/a	n/a
Young (2018)	USA	2016–2017	Cross-sectional	Hospital	Process indicators	n/a	n/a
Antonello (2019)	Brazil	2010–2015	Cross-sectional	Hospital	Adherence to guidelines	99	107
Yoon (2019)	New Zealand	2016	Before-after	Hospital	Process indicators, adherence to guidelines	1041	1064

N/A: not available; n/a: not applicable.

**Table 2:** App characteristics.

Author	App name	Custom built	Operating System	Content of app	Clinical indiction	Standalone	Interactive / static
Charani (2013)	IAPP	Yes	iOS & Android	Local therapeutic antimicrobial guidelines, calculator	Any infectious disease listed in guidelines	Yes	Interactive
Payne (2014)	iTreat	Yes	iOS	Local therapeutic antimicrobial guidelines & antimicrobial list	Any infectious disease listed in guidelines	Yes	Static
Panesar (2016)	MicroGuide	No	iOS, Android & WP	Local therapeutic antimicrobial guidelines & AMS section	Any infectious disease listed in guidelines	Yes	Static
Blumenthal (2017)	*N/A*	Yes	WEB-based	Local antimicrobial allergy guidelines	beta-lactam antibiotics for patients with listed penicillin allergy	Yes	Interactive
Charani (2017)	IAPP	Yes	iOS & Android	Local therapeutic antimicrobial guidelines, calculator	Any infectious disease listed in guidelines	Yes	Interactive
Fralick (2017)	*N/A*	Yes	iOS & Android	Local therapeutic antimicrobial guidelines & susceptibility results	Any infectious disease listed in guidelines	Yes	Static
Haque (2017)	Rehydration Calculator	Yes	Android	Therapeutic WHO guideline, calculator	Diarrhea	Yes	Interactive
Hoff (2018)	MicroGuide	No	iOS, Android & WP	Local therapeutic antimicrobial guidelines, antimicrobial list, susceptibility results & renal dosing guidelines	Any infectious disease listed in guidelines	Yes	Static
Tuon (2017)	*N/A*	Yes	iOS & Android	Local therapeutic antimicrobial guidelines & susceptibility results	Any infectious disease listed in guidelines	No	Static
Shenouda (2018)	MicroGuide	No	iOS, Android & WP	Local therapeutic antimicrobial guidelines	Any infectious disease listed in guidelines	Yes	Static
Young (2018)	*N/A*	Yes	iOS & Android	Local therapeutic antimicrobial guidelines, antimicrobial list, susceptibility results, perioperative antibiotic prophylaxis & dose adjustment based on renal funcion guideline	>50 infectious diseases listed in guidelines	No	Interactive
Antonello (2019)	ATB Fêmina	Yes	iOS & Android	Local diagnostic & therapeutic antimicrobial guidelines	Pyelonephritis during pregnancy	Yes	Static
Yoon (2019)	SCRIPT	Yes	iOS & Android	Local therapeutic antimicrobial guidelines	CAP and UTI	Yes	Interactive

Custom built: built for the study; Standalone: not integrated into the EHR system; Interactive: includes interactive elements, such as decision trees or calculators; N/A: not available; WP: Windows phone.

### Study quality

Emphasis on app dissemination and use, impact of app use and user experience resulted in quantitative, qualitative and mixed methods study designs such as: uncontrolled before-after (five), controlled before-after (two), interrupted time series (one), cross-sectional (six) and qualitative studies (three). Quality was evaluated with the corresponding tools [20–24]. Study designs varied greatly due to different metrics studied, e.g. app dissemination and use, impact of app use and user experience. Overall, methodological study quality was considered low to moderate for the before-after, interrupted time series and cross-sectional studies [25–35] and moderate to high for qualitative studies [30, 35, 36] (S4 Text). In most studies participants and outcome assessors were not blinded. Furthermore, outcomes were usually measured at one time point. Finally, the amount of eligible physicians enrolled was generally unclear as well as the loss to follow-up because information on user retention was lacking.

### Process indicators

Seven observational studies [26, 28, 30, 32–35] reported analytics of app use (Table 3). Five studies [26, 30, 32–34] evaluated total number of downloads. All registered an increase of downloads during their study periods (3–14 months). A study that assessed an app containing all hospital antimicrobial guidelines recorded an increase in average monthly app accessions during a 29-month period [32]. In contrast, the monthly app accessions decreased over 3 months in the study of Yoon *et al*. [34] which assessed an app containing only two guidelines, the treatment of community-acquired pneumonia and urinary tract infection. Additionally, clinicians accessed the guidelines more frequently by app than by desktop in all three studies evaluating number of accessions [26, 28, 30]. One study reported a decrease in time spent per individual guideline over the course of the study possibly demonstrating familiarization with the app [32]. The most frequently accessed guidelines were those outlining treatment for respiratory, skin & soft tissue and genitourinary infections [26, 28, 32, 34].

**Table 3:** Process indicators.

Author	Downloads		Average monthly use		Individual sessions		Time used per feature/session		Accessed guidelines
	Initial	Total	Initial	Follow-up	App	Non-app	Initial	Follow-up	(most frequent to least frequent)
Charani (2013)	376 times in first month	990 times after 12 months	250–300 average monthly users	*N/A*	1900 monthly average individual sessions (89.6%)	221 average monthly individual sessions on the intranet version (10.4%)	*N/A*	*N/A*	*N/A*
Payne (2014)	*N/A*	*N/A*	*N/A*	*N/A*	*N/A*	*N/A*	Time spent per day on the antimicrobial formulary (users): 0 minutes (8), 1 to 10 minutes (16), 11 to 20 minutes (5) and 21 to 30 minutes (2). Time spent per day on management protocols (users): 0 minutes (9), 1 to 10 minutes (20), 11 to 20 minutes (2) and 21 to 30 minutes (0)	*N/A*	*N/A*
Hoff (2017)	*N/A*	3056 times over 14 months	*N/A*	*N/A*	9259 times in total (53.0%)	8214 times in total per web viewer (47.0%)	*N/A*	*N/A*	Community-acquired pneumonia (3725), Antibiogram—Gram-negatives (3216), Antibiogram Gram-positives (2931), Antimicrobial dosing in renal insufficiency (2918), Spontaneous bacterial peritonitis (2576), Uncomplicated cystitis (2139)
Tuon (2017)	*N/A*	1741	*N/A*	*N/A*	*N/A*	*N/A*	50% of all sessions < 1 min.	*N/A*	*N/A*
Panesar (2016)	*N/A*	2013 times over 10 months	1182 average monthly accessions in first year (range: 1005–1615)	1483 average monthly accessions in 19 months (range: 945–2140)	>16 000 times in total	*N/A*	12.5 seconds average per individual guideline in first year	10.6 seconds average per guideline 19 months	UTI (lower), Pneumonia, Cellulitis, UTI (upper/pyelonephritis), Sepsis
Young (2018)	*N/A*	*N/A*	1257–1953 sessions/month	*N/A*	18860 sessions on 1887 unique devices (per year) (79.8%)	4761 sessions (desktop) on 3151 desktops (per year) (20.2%)	Mean session duration: 2:22 min	*N/A*	UTI 336–688 sessions/month, RTI 329–596 sessions/month, SSTI 289–615 session/month, GI 108–195 sessions/month, genital infections 52–153 sessions/month
Yoon (2019)	53 times in first month	145 times after 3 months	21 average accessions per user in first month	12 and 11 average accessions per user in second resp. third month	*N/A*	*N/A*	CAP guideline: median of 11 seconds (IQR 7–17). UTI guideline: median of 18 seconds (IQR 12–29)	*N/A*	Respiratory (847), Skin and soft tissue (663), Gastrointestinal tract (500), Sepsis (467), Genitourinary (350), ENT (335), CNS (278)

The process parameters reported in evaluated studies. CAP: community-acquired pneumonia; CNS: central nervous system; ENT: ear, nose & throat; GI: gastrointestinal infection; IQR: inter quartile range; N/A: not available; RTI: respiratory tract infection; SSTI: skin and soft tissue infection; UTI: urinary tract infection.

### Adherence to guidelines

Four studies analysed whether empirical prescribing of antimicrobials was according to guidelines, such as choice of drug, dose, interval and route of administration [25, 31, 34, 37]. (Table 4) The study of Charani *et al*. in which all antimicrobial guidelines were implemented in the app reported a significant increase in adherence to guidelines in surgical wards and a non-significant increase in general medicine wards [37]. This increase persisted after six and twelve months. Two studies [25, 31] reported an significant increase in adherence to guidelines for pyelonephritis and uncomplicated diarrheal diseases respectively during the intervention period. One study [34] showed increased adherence to the community-acquired pneumonia guideline in one hospital, but not in the other. Also, no change in adherence to the UTI guideline was shown in any of the three participating hospitals. Documentation of stop/review dates and indication for starting antimicrobials in the medical charts was evaluated in one study [37]. No change in documentation of stop/review dates was reported during or after the intervention period. Remarkably, documentation on the reason to start antimicrobials decreased significantly during intervention and this sustained during follow up measurements over the next two years [37].

**Table 4:** Adherence to guidelines.

Author	Country	Study duration		Number of patients included		Outcome	Collection of outcomes	Antimicrobial guideline(s)	Change in guideline adherent prescribing	
		Pre-intervention	Intervention	Pre-intervention	Intervention				Baseline to intervention	Follow-up
Charani (2017)	UK	36 months	36 months	*N/A*	*N/A*	Choice of antimicrobial	Biannual PPS	All available hospital guidelines	Medicine: 6.48% increase, 95% CI = –1.25 to 14.20 Surgery: 6.63% increase, 95% CI = 0.15–13.10, p<0.05	Effect positive after 6 and 12 months
Haque (2017)	Bangladesh	1.5 months	1.5 months	325	516	Choice of antimicrobial	Continuous measurement	Diarrhea	District hospital: 13% to 87%, p < 0.001 Sub-district hospital: 63% to 99%, p = 0.35	*N/A*
Antonello (2019)	Brazil	7 months	11 months	99	107	Choice of antimicrobial, dosage, Interval, route of administration	Continuous measurement	Pyelonephritis during pregnancy	Appropriate choice of antimicrobial drug 83.8% to 100%, p < 0.001; Appropriate dosage 100% to 100%, p = 1; Appropriate route of administration 97.0% to 100%, p = 0.018; Appropriate interval 91.9% to 100%, p = 0.004	*N/A*
Yoon (2019)	New Zealand	5 months	3 months	1041	1064	Guideline adherherence based on: Choice of antimicrobial, dosage, route of administration	Continuous measurement	CAP and UTI	CAP: Hospital 1: 19% to 27%, p = 0.04; Hospital 2: 9% to 9%, p = 0.98 UTI: Hospital 1: 47% to 50%, p = 0.49; Hospital 2: 45% to 40%, p = 0.28; Hospital 3: 24% to 29%, p = 0.25	*N/A*

Adherence to guidelines parameters reported in evaluated studies. CAP: community-acquired pneumonia; N/A: not available; PPS: point prevalence study; UTI: urinary tract infection

### User experience

In five studies user experience was analysed by means of interviews, focus groups or surveys [27, 30, 32, 35, 36]. The app was considered easy to use by 77.4% [35], 88.9% [27] and >90.0% [32] and useful by 71.0% [35], 85.2% [27] and >90.0% [32] of the users in before-after surveys with 31, 27 and 112 respondents, respectively. In one survey, 59 respondents reported app use increased their knowledge base regarding antimicrobial prescribing, while 81% reported app use helped them adhere to the guidelines [30]. Another survey reported 68% of the 31 respondents found app use time saving [35] Interviewees [36] as well as >90% of 112 survey respondents [32] favoured the app guidelines over the web viewer or paper guidelines. Discomfort using the app in front of patients or colleagues due to a sense of unprofessionalism was mentioned by 20.0% of 59 survey respondents [30] and 35.7% of 14 interviewees [36] but this was not experienced by others [32].

Frequent app use was inversely associated (survey respondents (SR) 106; risk ratio (RR) 0.03; confidence interval (CI) 0.0018–0.5; p = 0.0002) with preferring senior physician advice over antimicrobial guidelines, while frequent app use encouraged users to discuss incorrect prescribing by colleagues (SR 92; RR 3.8; CI 1.5–9.7; p = 0.005) [32]. Furthermore, app use was associated with a 1.1 point (p = 0.04) higher change in knowledge score of antimicrobial prescribing in 62 medical students and junior physicians compared to the control group [27] and improved awareness of antimicrobial stewardship (SR 91; RR 6.8; CI 2.1–21.7; p = 0.001) [32].

### Drug consumption, susceptibility and costs

Monthly average antimicrobial drug consumption was the focus of one study conducted in Brazil [33]. The app used in this study contained guidelines advising against use of some antimicrobials (e.g. carbapenems) while encouraging use of others (i.e. aminoglycosides) based on cost and susceptibility profile. After app introduction, the use of aminoglycosides and cefepime increased significantly while the use of piperacillin/tazobactam and meropenem decreased significantly. However, it should be noted that during the study period piperacillin/tazobactam was replaced by cefepime within the guideline for hospital-acquired infections. Furthermore, a significant increase in susceptibility to meropenem (73%–83%, p < 0.05) and polymyxin (69%–83%, p < 0.05) and a significant decrease in susceptibility to cefepime was described post-intervention (62%–57%, p < 0.05) [33]. In the year of implementation, a significant reduction of $296,485 USD (p<0.05) in antimicrobial drug costs was attained compared to the pre-implementation year. In a different study the optimal approach to promote safe use of beta-lactam antibiotics in inpatients with a history of penicillin allergy was evaluated [29]. Penicillin and cephalosporin use increased in the intervention period after introduction of a decision support app containing a decision tree for beta-lactam antibiotics (50% of 199 patients) compared to the standard of care period (38% of 148 patients). In the app intervention period odds of treatment with penicillin and cephalosporin were significantly increased (aOR 1.8% [95% CI 1.1, 2.9]).

## Discussion

In this systematic review, 13 studies which assessed antimicrobial stewardship smartphone apps in the hospital setting were analysed. Several studies measured different outcomes, applied different designs and varied in quality. In the reviewed studies, AMS apps were increasingly used or downloaded after implementation in five studies, guideline adherent prescribing of antimicrobials increased overall significantly in four studies and in one study this resulted in significantly less resistance to some antimicrobials and to a significant decrease in total drug costs. In general, users favoured the app based guidelines over web or paper versions in two studies, but some reported that app use in front of patients or colleagues felt unprofessional in three studies. Overall, although of varying quality, the studies indicate that AMS apps might increase guideline accessibility and offer physicians a friendly and efficient way of using antimicrobial guidelines.

Content of all but one app in the reviewed studies focused solely on therapy to improve the prescribing of antimicrobials according to the guidelines. To evaluate appropriate prescribing of antimicrobials, different outcome parameters were selected: choice of antimicrobials, dose, dosing interval and route of administration. One study also included indication and stop/review date documentation [37]. This variation in outcome parameters reflects the difficulty in defining appropriate antimicrobial prescribing. Since Gyssens *et al*. first described quality indicators (QI) for appropriate antimicrobial prescribing in 1992, QI’s have been added and debated in the infectious diseases community without reaching consensus, although many different quality indicators have been proposed [38–40]. In the studies reviewed, a limited set of outcome parameters was evaluated, leaving out many insightful quality indicators of appropriate antimicrobial prescribing, such as switch from intravenous to oral therapy and timely initiation of antimicrobial therapy [39]. Clearly defining quality indicators is essential in order to prevent interpretive bias and to compare studies promoting prescribing interventions.

A factor that was not always taken into account but should be considered for each AMS app is how users experience them. Several studies show that physicians with different backgrounds are enthusiastic about apps, find them user-friendly and helpful in their work and would recommend them to colleagues [41, 42]. In the studies reviewed, some physicians regarded smartphone use unprofessional in front of patients. However, a study focussing on outpatients found that more than half of patients were “fine” when physicians used their smartphone to access information during consultations, and thirteen percent reported it was “not fine” [43]. Initially used for calling and messaging, the increasing number of features and applications helped evolve the mobile phone into a possible valuable multifunctional tool for personal and professional use.

AMS apps could help to educate students, but also junior and senior physicians, in antimicrobial use. Although for students the education on antimicrobials and AMS varies within and between countries, globally, the knowledge of appropriate antimicrobial prescribing of final-year medical students is limited [44–47]. Additionally, prescribing errors are prevalent among junior physicians who are usually responsible for the majority of drug prescriptions in hospitals [48, 49]. The reviewed studies showed that AMS apps have additional educational value by improving knowledge of medical students and junior physicians on the prescribing of antimicrobials. Furthermore, as some of the younger smartphone using doctors have become attending physicians, smartphones will be an increasingly used tool for the prescription of antimicrobials.

The effect of clinical decision support systems (CDSS) on antimicrobial prescribing in the healthcare setting such as hospitals have been evaluated in many studies including several randomized controlled trials and systematic reviews [50–52]. Many are designed to improve guideline adherent prescribing of antimicrobials and are integrated into the EHR. Alternatively, almost all studied apps in our systematic review are standalone facilitating easy implementation in hospitals, are low cost and pose no risk in regard to losing patient data. However, a standalone system lacks patient specific data such as allergies, lab results, microbiological test results and previous treatment with antimicrobials which are mandatory to assess before antimicrobials can appropriately be prescribed [52]. Overall, CDSS have a positive effect on adherence to guidelines for antimicrobial treatment and decreased antimicrobial consumption. Yet, similar to our findings, these outcomes were not unanimous [50, 51]. Furthermore, the studies we reviewed lacked important clinical outcomes reported in the studies evaluating CDSS such as mortality, length of stay and time to therapy. As for AMS apps user experience is an important stepping stone for successful CDSS implementation. In spite of many studies on CDSS and its impact on antimicrobial prescribing the need for good quality studies remains for this IT-intervention too [50–52].

### Strengths & limitations

This systematic review has some strengths and limitations. One of the strengths is the clear start date of studies on this subject which coincides with the date app stores launched. A limitation is the exclusion of studies focusing on general practice or outpatient care. Therefore, we cannot draw conclusions on the advantage of AMS app use in these settings. Since the overall methodological study quality varied considerably and comparison between studies was limited due to large variations in study design and outcome parameters, only cautious conclusions could be drawn. Currently, the impact of a smartphone app on antimicrobial prescribing by physicians in hospitals is being evaluated in an international randomized trial [53].

## Conclusions

In this systematic review, the crossroad of healthcare and smartphone technology was explored. Smartphones may be used to improve knowledge of antimicrobial stewardship, to access antimicrobial guidelines and thereby improve important aspects of healthcare. During implementation of AMS apps, physician opinions and app uptake should be considered to optimize its impact. The small number of studies on AMS apps illustrate the novelty of this research area. Additionally, the quality of the data was limited. High quality, randomized, multi-centre studies including robust clearly defined clinical, microbiological and process outcomes are needed to evaluate the impact of AMS apps on antimicrobial prescribing and its role within healthcare.

## Supporting information

S1 Text PRISMA checklist. (DOC)

S2 Text Search strategy. (DOC)

S3 Text Data extraction form. (DOC)

S4 Text Quality assessment. (DOCX)
