# Supplementary material for: Use of stewardship smartphone applications by physicians and prescribing of antimicrobials in hospitals: A systematic review
Source: PLoS One. 2020 Sep 29;15(9):e0239751. doi: 10.1371/journal.pone.0239751 (PMC7523951; doi:10.1371/journal.pone.0239751)
Supplement: S2 Text — (DOC) [file pone.0239751.s002.doc]

**Search strategy**

Results

| *Database* | *Number of refs* | *Refs after deduplication* |
| --- | --- | --- |
|  |  |  |
| Embase.com | 549 | 539 |
| Medline (Ovid) | 184 | 42 |
| Cochrane Central | 36 | 20 |
| Web of Science | 151 | 47 |
| Google Scholar | 100 | 78 |
|  |  |  |
| **Total** | **1020** | **726** |

*Deduplicated: 249*

***Embase.com: 549***

**(**'antiinfective agent'/exp *OR 'antimicrobial stewardship'/de OR 'antimicrobial stewardship program'/de* OR (((anti) NEXT/3 (infecti* OR microb* OR bacter* *OR parasit* OR protozoa* OR malaria** OR virus OR viral OR bioti* OR fung* OR myco* OR spiroch*)) OR ((microb* OR bacter*) NEXT/3 (agent* OR drug*)) OR antiinfecti* OR antimicrob* *OR microbicide* OR antiparasit* OR antiprotozoa* OR antimalari** OR antibact* OR antifung* OR antimyco* OR antibioti* OR antispiroch* OR antivirus OR antiviral *OR stewardship**):ab,ti**) AND (**'prescription'/de *OR 'antimicrobial stewardship'/de OR 'antimicrobial stewardship program'/de* OR 'decision making'/de OR 'practice guideline'/de OR (prescri* *OR stewardship* OR guideline* OR (decision AND making)*):ab,ti**) AND (**'mobile phone'/exp OR 'mobile application'/exp OR (((mobile OR smart OR cell* OR portabl* OR computer* OR android *OR digital* OR portable*) *NEAR/4* (phone* OR telephon* OR app OR apps OR application* OR software* OR device* OR tablet* *OR assistant*)) OR smartphone* OR cellphone* OR iPhone* OR ipad* *OR handheld* OR hand-held**):ab,ti**)**

***Medline Epub: 184***

**(**exp Anti-Infective Agents/ OR (((anti) ADJ3 (infecti* OR microb* *OR parasit* OR protozoa* OR malaria** OR bacter* OR virus OR viral OR bioti* OR fung* OR myco* OR spiroch*)) OR ((microb* OR bacter*) ADJ3 (agent* OR drug*)) OR antiifecti* OR antimicrob* *OR microbicide* OR antiparasit* OR antiprotozoa* OR antimalari** OR antibact* OR antifung* OR antimyco* OR antibioti* OR antispiroch* OR antivirus OR antiviral *OR stewardship**).ab,ti.**) AND (**exp Drug Prescriptions/ OR exp Decision Making/ OR Practice Guidelines as Topic/ OR (prescri* *OR stewardship* OR guideline* OR (decision AND making)*).ab,ti.**) AND (**Cell Phone/ OR Mobile Applications/ OR (((mobile OR smart OR cell* OR portabl* OR computer* OR android *OR digital* OR portable*) ADJ*4* (phone* OR telephon* OR app OR apps OR application* OR software* OR device* OR tablet* *OR assistant*)) OR smartphone* OR cellphone* OR iPhone* OR ipad* *OR handheld* OR hand-held**).ab,ti.**)**

***Cochrane Central (trials): 36***

**(**(((anti) NEXT/3 (infecti* OR microb* OR bacter* *OR parasit* OR protozoa* OR malaria** OR virus OR viral OR bioti* OR fung* OR myco* OR spiroch*)) OR ((microb* OR bacter*) NEXT/3 (agent* OR drug*)) OR antiifecti* OR antimicrob* *OR microbicide* OR antiparasit* OR antiprotozoa* OR antimalari** OR antibact* OR antifung* OR antimyco* OR antibioti* OR antispiroch* OR antivirus OR antiviral *OR stewardship**):ab,ti**) AND (**(prescri* *OR stewardship* OR guideline* OR (decision AND making)*):ab,ti**) AND (**(((mobile OR smart OR cell* OR portabl* OR computer* OR android *OR digital* OR portable*) *NEAR/4* (phone* OR telephon* OR app OR apps OR application* OR software* OR device* OR tablet* *OR assistant*)) OR smartphone* OR cellphone* OR iPhone* OR ipad* *OR handheld* OR hand-held**):ab,ti**)**

***Web of Science: 151***

**TS=((**(((anti) NEAR/2 (infecti* OR microb* OR bacter* *OR parasit* OR protozoa* OR malaria** OR virus OR viral OR bioti* OR fung* OR myco* OR spiroch*)) OR ((microb* OR bacter*) NEAR/2 (agent* OR drug*)) OR antiifecti* OR antimicrob* *OR microbicide* OR antiparasit* OR antiprotozoa* OR antimalari** OR antibact* OR antifung* OR antimyco* OR antibioti* OR antispiroch* OR antivirus OR antiviral *OR stewardship**)**) AND (**(prescri* *OR stewardship* OR guideline* OR (decision AND making)*)**) AND (**(((mobile OR smart OR cell* OR portabl* OR computer* OR android *OR digital* OR portable*) *NEAR/4* (phone* OR telephon* OR app OR apps OR application* OR software* OR device* OR tablet* *OR assistant*)) OR smartphone* OR cellphone* OR iPhone* OR ipad* *OR handheld* OR hand-held**)**)**)

***Google Scholar: 100*** *(top relevant refs)*

**"**anti infective|microbial"|"microbial|bacterial agents|drugs"|antimicrobial|antibacterial|antibiotics|antiviral|*stewardship* prescription|prescribing*|stewardship* **"**mobile|smart phone|app|apps|application|device"|smartphone|iPhone|ipad*|handheld*
