# Supplementary material for: Use of stewardship smartphone applications by physicians and prescribing of antimicrobials in hospitals: A systematic review
Source: PLoS One. 2020 Sep 29;15(9):e0239751. doi: 10.1371/journal.pone.0239751 (PMC7523951; doi:10.1371/journal.pone.0239751)
Supplement: S3 Text — (DOC) [file pone.0239751.s003.doc]

**Data extraction form**

**General**

First author

Year of publication

Journal

Country

**Methods**

Study design

Study period

Total study duration

Sequence generation

Blinding

Other concerns about bias

**Participants**

Setting

Total number of prescribers

Total number of patients

Targeted prescribing population

Targeted patient population

Diagnostic criteria

**Interventions**

Total number of intervention groups

Specific intervention

**Apps**

Existing or custom app

Operating system

Native or web app

Integrated or standalone

Content

Interactive features

**Outcomes**

Outcomes

Timepoints outcomes were collected

**Results**

Number of participants allocated to each intervention group

Results (per timepoint)

**Miscellaneous**

Funding source
