# Supplementary material for: Use of stewardship smartphone applications by physicians and prescribing of antimicrobials in hospitals: A systematic review
Source: PLoS One. 2020 Sep 29;15(9):e0239751. doi: 10.1371/journal.pone.0239751 (PMC7523951; doi:10.1371/journal.pone.0239751)
Supplement: S4 Text — (DOCX) [file pone.0239751.s004.docx]

Quality assessment for uncontrolled before-after studies according to the National Institute of Health’s Quality Assessment Tool (23)

| Study question or objective clearly stated | + | + | + | + | + |
| --- | --- | --- | --- | --- | --- |
| Eligibility/selection criteria for the study population prespecified and clearly described | + | - | + | + | + |
| The participants in the study representative of those who would be eligible for the test/service/intervention in the general or clinical population of interest? | + | + | + | + | + |
| All eligible participants that met the prespecified entry criteria enrolled? | - | ? | - | - | - |
| The sample size was sufficiently large to provide confidence in the findings | + | + | + | - | - |
| The test/service/intervention clearly described and delivered consistently across the study population | NR | ? | + | + | + |
| The outcome measures prespecified, clearly defined, valid, reliable, and assessed consistently across all study participants | + | + | - | + | - |
| The people assessing the outcomes blinded to the participants' exposures/interventions | - | - | ? | ? | ? |
| The loss to follow-up after baseline 20% or less. Those lost to follow-up accounted for in the analysis | + | NR | + | - | + |
| The statistical methods examined changes in outcome measures from before to after the intervention. Statistical were tests done that provided p values for the pre-to-post changes | + | + | + | - | - |
| Outcome measures of interest were taken multiple times before the intervention and multiple times after the intervention (i.e., did they use an interrupted time-series design) | - | + | - | - | - |
| If the intervention was conducted at a group level (e.g., a whole hospital, a community, etc.) the statistical analysis took into account the use of individual-level data to determine effects at the group level | + | NR | + | - | - |
|  | Haque et al. (2017) | Tuon et al. (2017) | Panesar (2016) | Payne (2014) | Charani (2013) |

+: yes -: no ?: unclear NR: not reported

≤33.33% points: high risk of bias; >33.33% - ≤ 66.66% points: moderate risk of bias; >66.66% points: low risk of bias

Supplement: Quality assessment for ITS studies according to the Cochrane Collaboration’s tool for assessing risk of bias (21)

| Intervention independent of other changes | - |
| --- | --- |
| Shape of the intervention effect prespecified | + |
| Intervention unlikely to affect data collection | + |
| Blinding of participants and personnel | - |
| Blinding of outcome assessment | ? |
| Incomplete outcome data adequately addressed | + |
| Reports of the study free of suggestion of selective outcome reporting? | + |
| Study free from other risks of bias | - |
|  | Charani et al. (2017) |

+: yes -: no ?: unclear

≤33.33% points: high risk of bias; >33.33% - ≤ 66.66% points: moderate risk of bias; >66.66% points: low risk of bias

Supplement: Quality assessment for controlled before-after studies according to the Cochrane Collaboration’s tool for assessing risk of bias (20)

| Random sequence generation | - | - |
| --- | --- | --- |
| Allocation concealment | - | - |
| Blinding of participants and personnel | - | - |
| Blinding of outcome assessment | ? | ? |
| Incomplete outcome data | - | + |
| Selective reporting | + | + |
| Other sources of bias | - | + |
|  | Fralick et al. (2017) | Yoon et al. (2019) |

+: yes -: no ?: unclear

≤33.33% points: high risk of bias; >33.33% - ≤ 66.66% points: moderate risk of bias; >66.66% points: low risk of bias

Supplement: Quality assessment for cross-sectional studies according to the National Institute of Health’s Quality Assessment Tool (24)

| Research question or objective in this paper clearly stated | + | + | + | + | + | + |
| --- | --- | --- | --- | --- | --- | --- |
| Study population clearly specified and defined | - | + | - | + | + | + |
| Participation rate of eligible persons at least 50% | NR | ? | ? | - | ? | ? |
| All the subjects selected or recruited from the same or similar populations (including the same time period)? Inclusion and exclusion criteria for being in the study prespecified and applied uniformly to all participants | ? | - | - | - | - | - |
| A sample size justification, power description, or variance and effect estimates provided | - | - | - | - | - | - |
| For the analyses in this paper, were the exposure(s) of interest measured prior to the outcome(s) being measured? | - | - | - | - | - | - |
| The timeframe sufficient so that one could reasonably expect to see an association between exposure and outcome if it existed | + | + | + | + | + | + |
| For exposures that can vary in amount or level, did the study examine different levels of the exposure as related to the outcome (e.g., categories of exposure, or exposure measured as continuous variable)? | - | - | - | - | - | - |
| The exposure measures (independent variables) clearly defined, valid, reliable, and implemented consistently across all study participants | - | - | - | + | - | + |
| The exposure(s) assessed more than once over time | NR | - | - | + | - | - |
| The outcome measures (dependent variables) clearly defined, valid, reliable, and implemented consistently across all study participants | - | + | - | + | + | + |
| The outcome assessors blinded to the exposure status of participants | NA | NA | - | - | NA | NA |
| Loss to follow-up after baseline 20% or less | ? | ? | ? | + | ? | ? |
| Key potential confounding variables measured and adjusted statistically for their impact on the relationship between exposure(s) and outcome(s) | - | - | - | + | - | - |
|  | Hoff et al. (2018) | Young et al. (2018) | Antonello et al. (2019) | Blumenthal et al. (2017) | Panesar et al. (2016) | Charani et al. (2013) |

+: yes -: no ?: unclear NR: not reported NA: not applicable

≤33.33% points: high risk of bias; >33.33% - ≤ 66.66% points: moderate risk of bias; >66.66% points: low risk of bias

Supplement: Quality assessment for qualitative (part of) studies according to the Critical Appraisal Skills Programme (CASP) Qualitative Checklist (22)

| Clear statement of the aims of the research | + | + | + |
| --- | --- | --- | --- |
| A qualitative methodology is appropriate | + | + | + |
| Research design appropriate to address the aims of the research | + | + | + |
| Recruitment strategy appropriate to the aims of the research | - | + | + |
| Data collected in a way that addressed the research issue | ? | ? | + |
| Relationship between researcher and participants adequately considered | - | ? | - |
| Ethical issues taken into consideration | ? | + | + |
| Data analysis sufficiently rigorous | ? | ? | ? |
| A clear statement of findings | + | - | + |
|  | Charani et al. (2013) | Payne et al. (2014) | Shenouda et al. (2018) |

+: yes -: no ?: unclear

≤33.33% points: high risk of bias; >33.33% - ≤ 66.66% points: moderate risk of bias; >66.66% points: low risk of bias
